# Integrated Physiological and Metabolomic Analyses of the Effect of Potassium Fertilizer on Citrus Fruit Splitting

**DOI:** 10.3390/plants11040499

**Published:** 2022-02-12

**Authors:** Yun Jiao, Cunlong Sha, Qiaoyun Shu

**Affiliations:** 1 Institute of Forestry, Ningbo Academy of Agricultural Science, Ningbo 315040, China; shuqiaoyun2022@163.com; 2 Haishu District Agricultural Technology Management Service Station, Ningbo 315040, China; zln306@163.com

**Keywords:** potassium, citrus, fruit splitting, physiology, metabolite

## Abstract

Potassium (K) nutrition plays a key role in alleviating a variety of peel disorders in tree fruit, but the effect of this nutrient on the physiological and metabolic profiles involved in the fruit splitting of citrus remains unclear. Three levels of K were used to treat citrus ‘Ehime Kashi 34’ (*Citrus* Nishinoka × *C.* Shiranui), a hybrid cultivar with fruit that easily split. The results showed that the roots of the treatment with K fertilizer increased the contents of calcium (Ca^2+^), nitrogen (N), and K in the skin and flesh, the fruit firmness ratio of the peel to the flesh, photosynthetic rate, stomatal conductance, and concentration of intercellular CO_2_. In contrast, it decreases the relative chlorophyll index and content of Ca^2+^ in the leaves. Simultaneously, 59 and 13 differentially expressed metabolites (DEMs) were detected in the peel and flesh, respectively, after treatment with K. Of them, five compounds were upregulated, including the synthesis of various amino acids in the peel and the accumulation of various glycoside metabolites in the flesh which were upregulated. The accumulation of levels of gibberellin and glycoside were downregulated. That could be the main reason why potassium alleviates fruit splitting.

## 1. Introduction

Late-maturing citrus varieties, such as ‘Ehime Kashi No. 28’ and ‘Ehime Kashi No. 34’, are increasing in economic importance owing to their preferable flavor and maturity period close to the Chinese New Year [1,2], Unfortunately, these varieties are susceptible to a disorder of the fruit peel, termed fruit splitting, which seriously affects the quality of fruit and causes substantial economic losses. Studies have indicated that fruit splitting is caused by a combination of environmental factors that trigger its occurrence with a genetic predisposition. These factors include a nutritional imbalance, low potassium (K) and calcium (Ca^2+^) and an irregular supply of water. In short, fruit splitting is a type of physiological disorder that is jointly owing to the expression of factors in the external environment and those that are internal in the fruit [3].

It is well known that K Is the most abundant mineral nutrient element in citrus fruits [4]. Many studies have shown that K restricts fruit size, soluble solids, and yield, as well as total fruit acidity (TA) and peel thickness [5,6,7]. It is worth noting that if K is augmented to easily cracked citrus cultivars to achieve a level of 1.0% to 1.5% of K in the leaves, the peel will be thicker and not easily split [3]. This could be owing to the ability of K to promote fruit cell division and root proliferation, increase the efficiency of root system to absorb nutrients important for plant growth, and increase the thickness and strength of the peel, thereby reducing fruit splitting [8,9]. However, the mechanisms of specific metabolic regulation are still unclear. In addition, K both directly and indirectly activates more than 120 enzymes that are involved in plant growth and development, including such areas as energy use, nitrogen metabolism, photosynthesis, and respiration, which, in turn, affect the level and composition of a wide variety of plant compounds [10,11,12]. Among them, primary metabolites, such as starch, soluble sugar, and amino acids, in rice (*Oryzae sativa*), soybean (*Glycine max*) and wheat (*Triticum aestivum*) are usually reduced when K is deficient in the soil root zone [13,14]. Alternatively, secondary metabolites in plants are also affected by the supply of K [15]. The total phenolics in blackberries decreased in parallel with the supply of K [16]. A deficiency in K can synthetically constrain growth owing to its effects on the diffusion of CO_2_ and assimilation products that will result in an impact on photosynthesis [17]. This results in a large accumulation of carbohydrates in the source leaves and limits the translocation of sucrose from buds to sink tissues, including fruit. This limit is thought to reduce the quality of fruit, such as tomato (*Solanum lycopersicum*). In contrast, when high levels of potassium, such as 270 kg K/ha, are used for fertilization, the activity of phenylalanine ammonia-lyase (PAL) in *Labisia pumila* increases, and the level of accumulation of total flavonoids, phenols and ascorbic acid increase, which will help improve the quality of fruit [16]. Therefore, K plays an important role during the process of regulating plant metabolites, but it is not enough to explain the specific effect of K on fruit splitting.

In short, potassium (K) has important physiological functions in the cultivation of most horticultural crops, and its effectiveness will also strongly affect the quality of citrus fruit [5,6,10]. Furthermore, owing to the unique anatomical characteristics of citrus fruits that differ from other crops, the intrinsic mechanism of supply of K on citrus fruit splitting has not been ascertained, and detailed studies on its effect on metabolites in fruits are still rare. In view of this, we used potted specimens of ‘Ehime Kashi No. 34’, a citrus variety that cracks easily as the material, and applied three K-level fertilization tests to study its effect on citrus fruit splitting (peel/flesh) and metabolite spectrum (non-targeted LC-MS), to comprehensively analyze the mechanism of K fertilizer on the fruit splitting of citrus by examining the physiology of its fruit and the contents of its metabolites.

## 2. Results

### 2.1. Effect of Potassium on the Quality of Citrus Fruit

**Figure 1:**
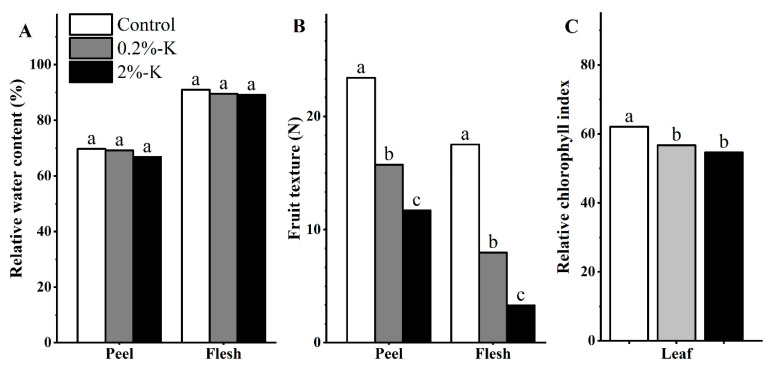
(**A**) The effect of different potassium levels on the relative water content of citrus fruits (peel/flesh). (**B**) The effect of different potassium levels on the texture of citrus fruit (peel/flesh). (**C**) The effect of different potassium levels on the leaf relative chlorophyll index of citrus. Significant differences (*p* < 0.05) were indicated by different lowercase letters according to the one-way ANOVA followed by a Duncan’s test.

As shown in Figure S1, the split forms of citrus fruit after K treatment can be divided into two types that include longitudinal and horizontal splitting, and the fruits were more likely to split horizontally than vertically (Table 1). Five percent of the fruits split after high K treatment. Therefore, K treatment could not completely prevent the fruit from splitting. In addition, the water content of the flesh of fruit was higher than that of the peel, and the difference in water content of the flesh/peel after different potassium treatments was not significant. However, the peel/flesh had the highest content of water after treatment with high K. In contrast, high K treatment resulted in a reduction of the fruit texture and relative chlorophyll index to the lowest levels (Figure 1). It is worth noting that the fruit hardness ratio was the highest after high potassium treatment (Table 1), which could be one of the important reasons for the low rate of fruit splitting.

### 2.2. Effect of Potassium on the Contents of Nutrient Elements and Photosynthesis in Citrus Fruits and Leaves

**Figure 2:**
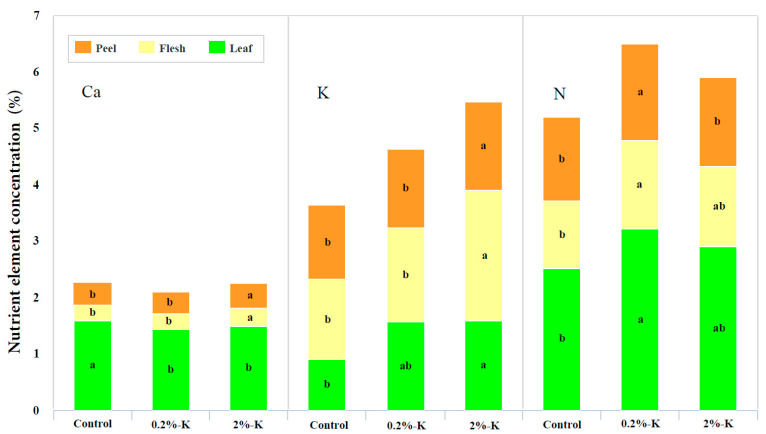
The effect of different potassium levels on the content of three nutrient elements (Ca^2+^, K, and N) in citrus peels, flesh and leaves. Significant differences (*p* < 0.05) were indicated by different lowercase letters according to the one-way ANOVA followed by a Duncan’s test.

As shown in Figure 2, the contents of Ca^2+^ and K in the fruit (peel and flesh) after treatment with 2%-K were higher than those of other treatments. However, the high K treatment resulted in a higher content of Ca^2+^ in the peel than in the flesh. In contrast, the content of K in the peel was lower than that in the flesh. Simultaneously, the content of K in the leaves was the highest after high K treatment, while the content of Ca^2+^ in the leaves was the highest in control group. This showed that the leaves are not the main tissues for Ca^2+^ storage following treatment with high K, and the fruit may play this role. Simultaneously, the content of N in the fruits and leaves after treatment with high K was lower than those following the 0.2%-K treatment. This pattern differs from that of Ca^2+^ and K. It is apparent that increasing the supply of K has different effects on the contents of Ca^2+^, K, and N in different tissues.

**Figure 3:**
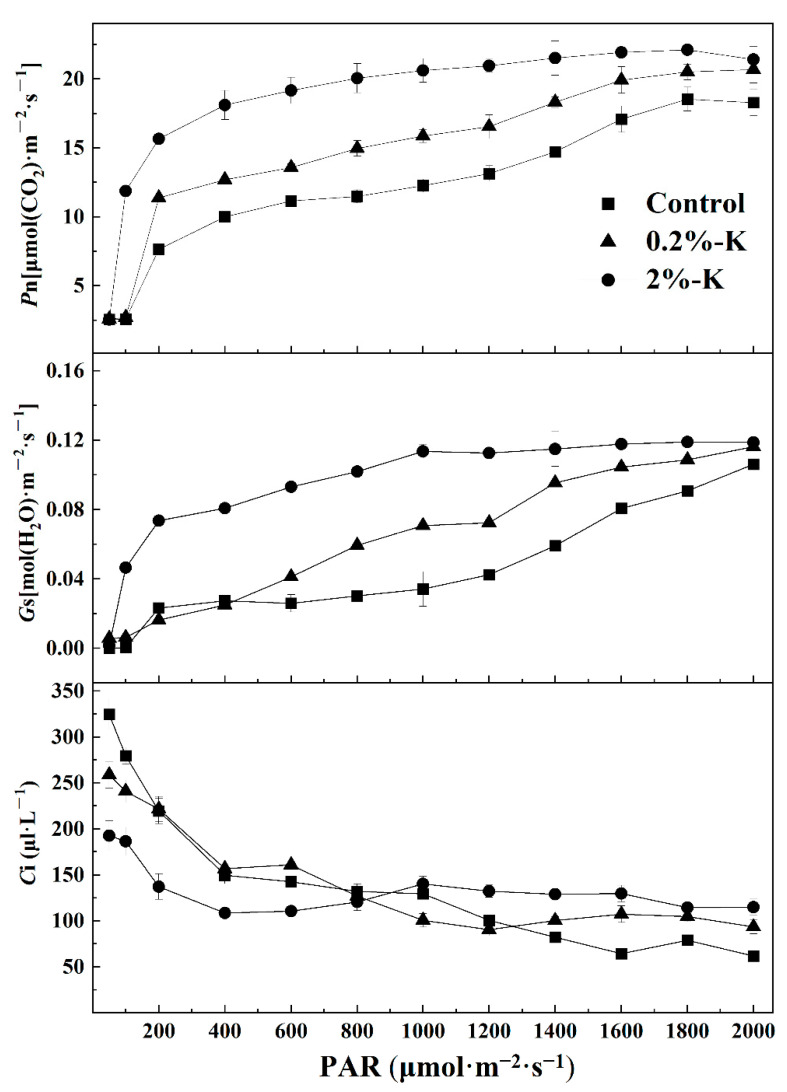
The effect of different potassium levels on the photosynthetic efficiency parameters in citrus. Photosynthetic rate (*P*n); stomatal conductance (*G*s); intercellular CO_2_ concentration (*C*i); photosynthetically active radiation (PAR).

In addition, the photosynthetic rate (*P*n) in leaves after the three K treatments all increased rapidly as the photosynthetically active radiation (PAR) increased (Figure 3). The upward trend of *P*n slowed and gradually reached saturation when the PAR was 1000–1800 μmol·m^−2^·s^−1^. At this time, the *P*n after high K treatment was similar to the trend of change in *G*s. In contrast, *C*i decreased slowly as the PAR increased. The *C*i value decreased to its lowest point when the PAR was 2000 μmol·m^−2^·s^−1^, and the high K treatment was still at its peak at this time. It is apparent that there are some differences in changes in the light response curve of leaves after different treatments with K. Therefore, increasing the amount of K could effectively improve the photosynthetic efficiency of citrus.

### 2.3. Effects of Potassium on the Components of Metabolites in Citrus Fruits

**Figure 4:**
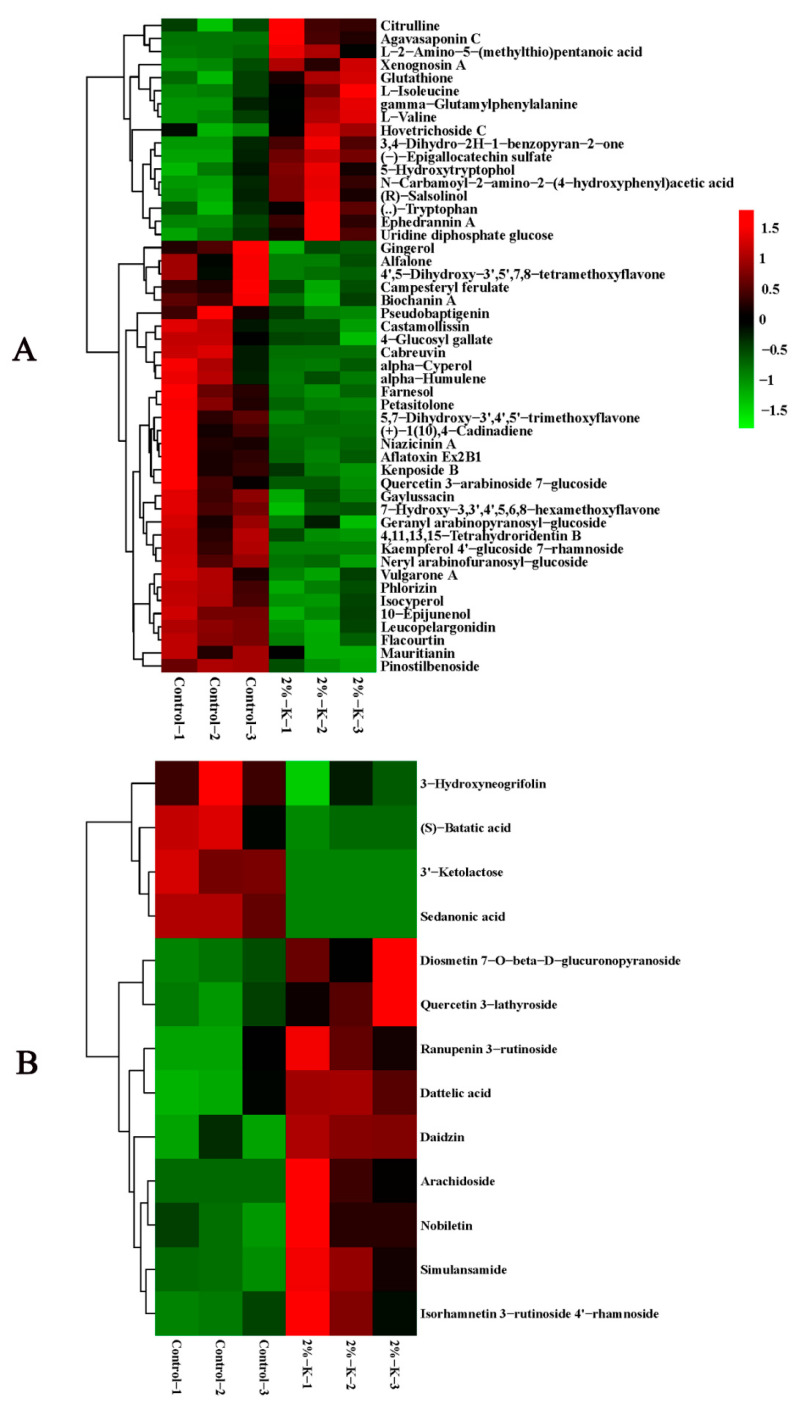
Hierarchical clustering heatmap of the top 50 DEMs in citrus fruit ((**A**), peel and (**B**), flesh) following treatment with 2% potassium. CK, control without potassium; 1–3, repetition. The levels of relative expression of the DEMs were normalized using log2. DEMs, differentially expressed metabolites.

**Figure 5:**
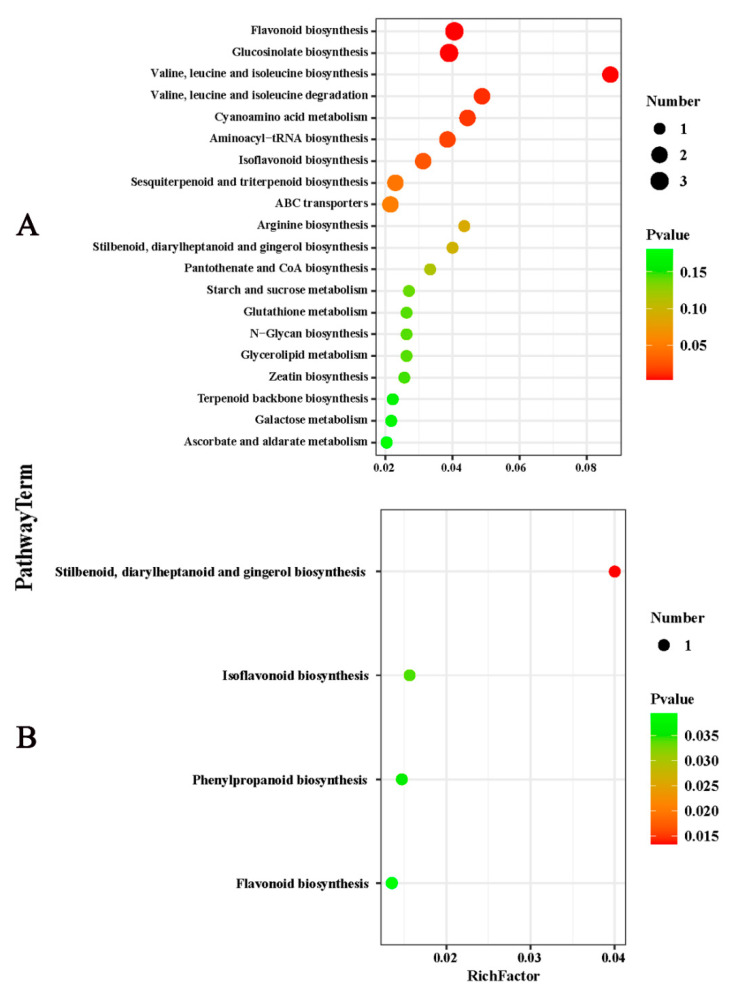
Top 20 enriched KEGG terms of citrus fruit ((**A**), peel and (**B**), flesh) following treatment with 2% potassium. The Rich factor indicates the degree of enrichment that is the *p* value after multiple hypothesis testing and ranges between 0 and 1. As *P* grows closer to zero, the enrichment becomes more significant. KEGG, Kyoto Encyclopedia of Genes and Genomes.

LC-MS identification and the OPLS-DA model with *p* < 0.05 and VIP > 1 indicated that the citrus peel contained 59 DEMs. Nineteen were upregulated, and the forty remaining DEMs were downregulated. These metabolites primarily included lipids and lipid molecules, phenylpropanoids and polyketides, organic acids and secondary metabolites. There were fewer DEMs in the flesh than in the peel, with only 13 that were upregulated and four that were downregulated. The DEMs primarily included phenylpropanoids and polyketides, organic oxygen compounds, and organic acids and their derivatives. The top 50 DEMs were analyzed through hierarchical clustering, and the heat map is shown in Figure 4A,B. The related pathways of all of the DEMs affected by K were investigated in the Kyoto Encyclopedia of Genes and Genomes (KEGG) database. The top 20 pathways rich in the peel DEMs included starch and sucrose metabolism, flavonoid biosynthesis, multiple amino acid metabolism, and endogenous hormone synthesis. However, only four pathways were enriched in the flesh, including flavonoids, isoflavones and benzene, and phenylpropanoid biosynthesis (Figure 5A,B). In short, the changes in these metabolic pathways and metabolites provide important information to explain the mechanism of K fertilizer on fruit splitting in citrus.

### 2.4. Overview of the Core DEMs in Citrus Fruit following Potassium Treatment

**Figure 6:**
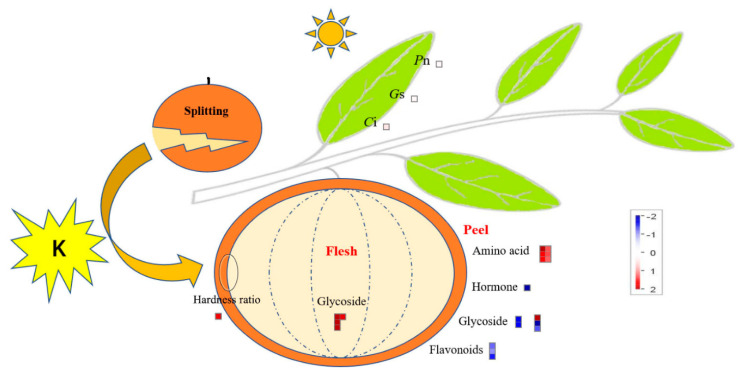
Overview of the citrus leaf photosynthesis and DEMs in the peel and flesh, which could involve the use of potassium to alleviate fruit splitting. The data in the figure is drawn from the results of metabolomics testing after potassium treatment (2%-K) in this study and normalized using log2. The physiological parameters of citrus leaf (PAR at 1800 μmol·m^−2^·s^−1^) include the photosynthetic rate (*P*n), stomatal conductance (*G*s), and intercellular CO_2_ concentration (*C*i). DEMs, differentially expressed metabolites.

MapMan was used to show the key factors in citrus leaf photosynthesis and fruit (peel/flesh) DEMs to clearly understand the mechanism of K fertilizer used to alleviate fruit splitting (Figure 6). As a result, 19 metabolites were located, including those involved in amino acid synthesis and metabolism, glycoside synthesis, synthesis and degradation of endogenous hormones, and the synthesis of flavonoid metabolites (Table S1). Among them, there were more metabolites in the peel, and they were more complex than those in the flesh. The high K treatment led to an increase in the accumulation of amino acid metabolites in the peel and glycoside metabolites in the flesh. Simultaneously, it led to an increase in the photosynthetic efficiency parameters (*P*n, *G*s, and *C*i). In contrast, this treatment caused endogenous hormones and glycoside metabolites (except ‘Agavasaponin C’) to accumulate, while flavonoid metabolites in the peel decreased. It is worth noting that the main endogenous hormone in the peel was gibberellin (log_2_FC = −30.02), but it was not detected in the flesh and may be one of the key factors to alleviate the splitting of the fruit.

## 3. Discussion

The thin peel of citrus hybrids increases their susceptibility to fruit splitting [18,19]. Most fruit splits start at the style or umbilicus end of the citrus peel, which could be since this area of the peel is thinner than that of other areas [20]. Another possibility is that the local stress caused by the expansion of the fruit inside is not balanced, and thus splits longitudinally. However, in comparison, the proportion of citrus that split horizontally was higher than that that split vertically (Table 1). Since this is not a single vertical division of the fruit, this special form of fruit splitting could be caused by factors specific to this variety, and this tendency to split is one of the characteristics of oval-shaped fruit citrus varieties. It is worth noting that since the fruit splitting is the result of the synergistic effect of the turgor pressure changes of the flesh and the peel [21,22], the fruit hardness ratio proposed in this study avoids conflating the peel and flesh. It is not advantageous to simply analyze the changes in peel hardness. By explaining the internal mechanism of fruit splitting, this study also revealed that K increases the hardness ratio of fruit, which could be a key factor that determines the tendency of fruit to split. Therefore, it should be considered to be one of the key research directions for the future. Unfortunately, fruit splitting cannot be completely avoided even after treatment with high K. However, further design experiments are merited to verify this analysis.

Similar to previous reports, treatment with high K increased the accumulation of primary metabolites (amino acids) in the peel [16,23]. Among the DEMs that were associated with the upregulation of accumulation of DEMs in the peel, six were amino acids, which may accelerate the accumulation of assimilates and have led to a difference in the cell water potential between the peel and the flesh. Thus, the peel can absorb sufficient water by osmosis and become more resilient. Ultimately, this reduces the susceptibility of the peel to splitting. Intriguingly, after the peel was treated with high K, the level of accumulation of 5-hydroxytryptophol increased. This is of note due to the fact that it can be used as a precursor of serotonin in the human body. Thus, it is thought to help increase the levels of serotonin, which plays an important role in the treatment of depression in humans [24]. However, further exploration is needed to study the regulatory mechanism and function involved in fruit splitting. In addition, gibberellin is a plant hormone that is responsible for promoting cell division and expansion [25,26]. Thus, spraying gibberellin is an important technical measure to increase fruit size, firmness, peel strain and reduce the fruit splitting rate in many woody fruit plants, such as grape (*Vitis vinifera*) and cherry (*Prunus avium*) [27,28]. This treatment usually negatively correlates with the splitting rate. However, unexpectedly, the level of accumulation of gibberellin in the peel after the high K treatment (low fruit splitting rate) in this study did not increase but instead decreased (Figure 6). This could be owing to the sampling time that took place during the second peak of fruit development when the volume of flesh increased, and the fruit need to consume more endogenous cytokinin, resulting in a decrease in the level of accumulation. Simultaneously, the citrus peel became more metabolically active following treatment with high K, and it may also consume an excessive amount of endogenous gibberellin, resulting in a decrease in the level of accumulation. Thus, there could be a direct or indirect interactive effect between K and gibberellin. Eventually, the balance of gibberellin in the flesh and peel will be disrupted, which will affect the incidence of fruit splitting. However, in-depth exploration and analysis are merited. In addition, relative to the peel, the richness of differential metabolites in the flesh was much lower with only 13 compounds. Therefore, the strain and toughness of the peel are more important to study to better understand fruit splitting.

In addition, treatment with high levels of exogenous of K activates biological pathways, such as isoleucine biosynthesis and degradation, flavonoid biosynthesis, cyanoamino acid metabolism and phenylpropanoid metabolism pathways. Previous studies have confirmed that they play an important role in the responses of plants to stress and control of the ion balance [29,30,31]. These findings will help to improve the stress resistance of citrus through the application of K fertilizer, thereby forming effective cultivation management measures [11]. Furthermore, previous studies have shown that putrescine in tomato leaves, as a potential biomarker metabolite, are strongly and negatively correlated with the concentration of K. However, this study did not find that putrescine exists as a differential metabolite. This could be caused by differences in the species, tissues, or organs. It is not suitable for citrus as a biomarker metabolite, and further research and analysis are merited.

It is worth noting that K is not the only factor that determines cell division in fruit. In addition, Ca^2+^, boron, and other elements are all involved in the regulation of fruit texture characteristics and have some effect on fruit splitting [32,33,34]. However, current research still primarily focuses on the mechanism of regulation of a single mineral element in the fruit developmental process, and the synergistic mechanism of two or more elements is rarely considered. Therefore, follow-up research should focus on exploring the mechanism of cooperative regulation of multiple mineral elements in fruit division. Simultaneously, based on the conclusions of this research, additional focus should be on exploring hormones, such as gibberellin, in the peel and flesh structure. The influence of synthesis and regulatory pathways on the growth rate and coordination of the peel and flesh will be more advantageous to comprehensively reveal the mechanism of fruit splitting.

## 4. Materials and Methods

### 4.1. Plant Treatment and Sampling

Four-year-old citrus grafted ‘Ehime Kashi No. 34’ (*C*. Nishinoka × *C.* Shiranui) plants were transplanted into plastic pots that were 38 cm diameter × 39 cm deep on 15 January 2021, in Ningbo City of Zhejiang Province, China (121°36′ N, 29°40′ E). The pot was pre-filled with 10 cm deep vermiculite to facilitate drainage, and the upper part (about 28 cm deep) was filled with clean river sand. A glass greenhouse was used under a natural light cycle and with a natural ventilation system for air exchange inside and outside the greenhouse. The building was isolated with gauze to avoid the invasion and spread of external pests and diseases. Fifteen pots in this study were cultivated for each treatment, and five pots were considered a replicate. Different nutrient solutions (control, 0.2%-K and 2%-K) were poured into the soil in three treated pots at an interval of one month, and the soil water content was maintained at 40 ± 5% with drip irrigation until sampling, using a soil moisture and temperature recorder (L99-TWS-1, Fotel Precise Instrument Co., Ltd., Shanghai, China). In short, the control, without K that consisted of a modified Hoagland solution (4 mM Ca(NO_3_)_2_, 2 mM NH_4_PO_4_, 1 mM MgSO_4_, 0.002 mM MnSO_4_,0.002 mM ZnSO_4_, 0.05 mM KCl, 0.025 mM H_3_BO_3_, 0.0005 mM CuSO_4_, 0.0005 mM H_2_MoO_4_, and 0.07 mM FeSO_4_, pH 6.8) [35]; 0.2%-K, the modified Hoagland solution (same as the control) that contained 0.2% K_2_SO_4_; and 2%-K: the modified Hoagland solution (same as the control) that contained 2% K_2_SO_4_, were applied as the three K treatment levels (0 K, 0.2%-K and 2%-K) in a randomized complete block design. A total of 15 fruit were collected for each treatment during the peak period of fruit splitting on 15 September 2021. Next, the fruit samples were used to determine the fruit texture, relative water content and the concentration of nutrient elements, and the peel and flesh were quickly separated and stored at −80 °C for non-targeted metabolomics.

### 4.2. Measurements of Physiological Characteristics

A Dualex 4 (FORCE-A, Orsay, France) was used to measure the relative chlorophyll index of citrus leaves using the nitrogen balance index (NBI) [36], and 20 leaves were measured for each treatment. The photosynthetic rate (*P*n), stomatal conductance (*G*s), and concentration of intercellular CO_2_ (*C*i) were measured at 10:00–11:00 am (15 September 2021) using an LI-6400XT portable photosynthetic rate meter (LI-COR Biosciences, Lincoln, NE, USA) for functional leaves, and 15 leaves were measured for each treatment. The light response curve measurement parameters included light intensities from 2000, 1800, 1600, 1400, 1200, 1000, 800, 600, 400, 200, 100 and 50 μmol·m^−2^·s^−1^. The CO_2_ content was set to 500 μmol·mol^−1^, and the LightCurve2 program [37] was used to automatically determine this data, which was compiled to draw the light response curve.

In addition, a TA.XT plus texture analyzer (Stable Micro System. Godalming, UK) was used to perform a texture profile analysis (TPA) of the equatorial axis tissue (peel/flesh) of the fruit as previously described [38]. The relative moisture content of the citrus peel and flesh was measured by weighing. The samples were first dried for two hours in a blast dryer at 105 °C, adjusted to 60 °C and dried to a constant weight. Microwave digestion and atomic absorption (AAS) were performed using an atomic absorption spectrophotometer (AA800; PerkinElmer, Waltham, MA, USA) to estimate the concentrations of K and Ca^2+^ in the fruit and leaves using 15 leaves for each treatment as previously described [39], and the N content was analyzed by an automatic nitrogen analyzer using the Kjeldahl method [40,41]. All of the data were exported to Microsoft Excel 2016 (Redmond, WA, USA), and an analysis of variance (ANOVA) by Duncan’s test (*p* < 0.05) was conducted using SPSS v. 15 (SPSS Inc., Chicago, USA). Finally, OriginPro software version 2021 (OriginLab, Northampton, MA, USA) was used for drawing.

### 4.3. LC/MS Untargeted Metabolomics Analysis

The sample was thoroughly mixed, and 8 mg from each replicate was combined with 20 μL of internal standard dissolved in methanol (0.3 mg/mL L-2-chlorophenylalanine and 0.01 mg/mL Lyso PC17:0) [42], and 1 mL of methanol: water (7:3 [*v*/*v*]) was added. Two small steel balls were then added, and the sample was pre-cooled at −20 °C for 2 min. It was then ground for 2 min at 60 Hz. The sample was extracted ultrasonically for 30 min before incubation at −20 °C for 20 min and then centrifugation at 14,000× *g* for 10 min at 4 °C. A volume of 300 µL of the supernatant was dried by evaporation, reconstituted with 400 μL methanol-water (1:4 [*v*/*v*]), vortexed for 30 s, ultrasonicated for 2 min, and centrifuged for 10 min at 14,000× *g* at 4 °C before 150 μL of the supernatant was withdrawn with a syringe. The supernatant was then filtered through a 0.22 μm organic phase pinhole filter and stored at −80 °C. An LC/MS analysis was performed using a liquid mass spectrometry system composed of a Dionex U3000 ultra-high performance liquid phase (UHPL) tandem QE Plus high-resolution mass spectrometer (ThermoFisher Scientific, Waltham, MA, USA). The data were collected in full scan mode (m/z range from 70 to 1000) combined with the information-related collection mode. The raw data were processed using the metabolomics processing software Progenesis QI v2.3 (Nonlinear Dynamics, Newcastle, UK) for baseline filtering, integration, peak identification, peak alignment, retention time correction, and normalization. The main parameters included the precursor tolerance 5 ppm, product tolerance 10 ppm, and product ion threshold 5%. The compounds were identified based on accurate mass, secondary fragments, and isotope distribution using the Human Metabolome Database (HMDB), Lipidmaps (v2.3), and the METLIN database for qualitative identification. Principal component analysis and (orthogonal) partial least square discriminant analysis (O) PLS-DA were used to examine the samples. The variable importance in projection (VIP) ranks the overall contribution of each variable to the OPLS-DA model, and variables with VIP > 1 and *p* < 0.05 are considered to be related to group discrimination [43]. In addition, MapMan version 3.6.0 (http://mapman.gabipd.org/web/guest, accessed on 15 November 2021) [44] was used to locate and analyze the DEM pathways.

## 5. Conclusions

This study shows that the roots of the treatment with K fertilizer can effectively alleviate the incidence of citrus fruit splitting. In addition, high K can promote the metabolism of amino acids in the peel of citrus fruit and accelerate the consumption of gibberellin, which may result in less fruit splitting. Treatment with K has little effect on the metabolites in flesh compared with the peel, indicating that the latter is more sensitive to K and a more prominent area in which to study fruit splitting. These results are highly significant for revealing the regulatory mechanism of K in the splitting of citrus fruit and will provide guidance for the scientific application of K in citrus production. However, the potential molecular genetic mechanism by which K alleviates the incidence of citrus fruit splitting merits further clarification.

## Figures and Tables

**Table 1:** The effect of different levels of potassium vertical and horizontal splitting in citrus ‘Ehime Kashi 34’ (*Citrus* Nishinoka × *C*. Shiranui) fruit. The hardness ratio refers to the ratio of the hardness of the peel to the flesh. Significant differences (*p* < 0.05) were indicated by different lowercase letters according to the one-way ANOVA followed by a Duncan’s test.

Treatment	Total Fruit Split (%)	Vertical (%)	Horizontal (%)	Hardness Ratio
Control	31.0 a	12.0 a	19.0 a	1.34 c
0.2%-K	15.0 b	5.00 b	10.0 b	1.97 b
2%-K	5.00 c	2.00 c	3.00 c	3.53 a

## Data Availability

All data are included in the main text.

## Supplementary Materials

The following supporting information can be downloaded at: https://www.mdpi.com/article/10.3390/plants11040499/s1. Figure S1: Two main appearance characteristics of citrus fruit split (vertical and horizontal). The samples were collected at the peak of fruit splitting during fruit development; Table S1: The key DEMs may help to alleviate fruit splitting following treatment with potassium fertilizer.
