# Supplementary material for: Integrated Physiological and Metabolomic Analyses of the Effect of Potassium Fertilizer on Citrus Fruit Splitting
Source: Plants (Basel). 2022 Feb 12;11(4):499. doi: 10.3390/plants11040499 (PMC8877888; doi:10.3390/plants11040499)
Supplement: Supplementary file 1 [file plants-11-00499-s001.zip › plants-1578783-supplementary.pdf]

Supplementary material

Figures

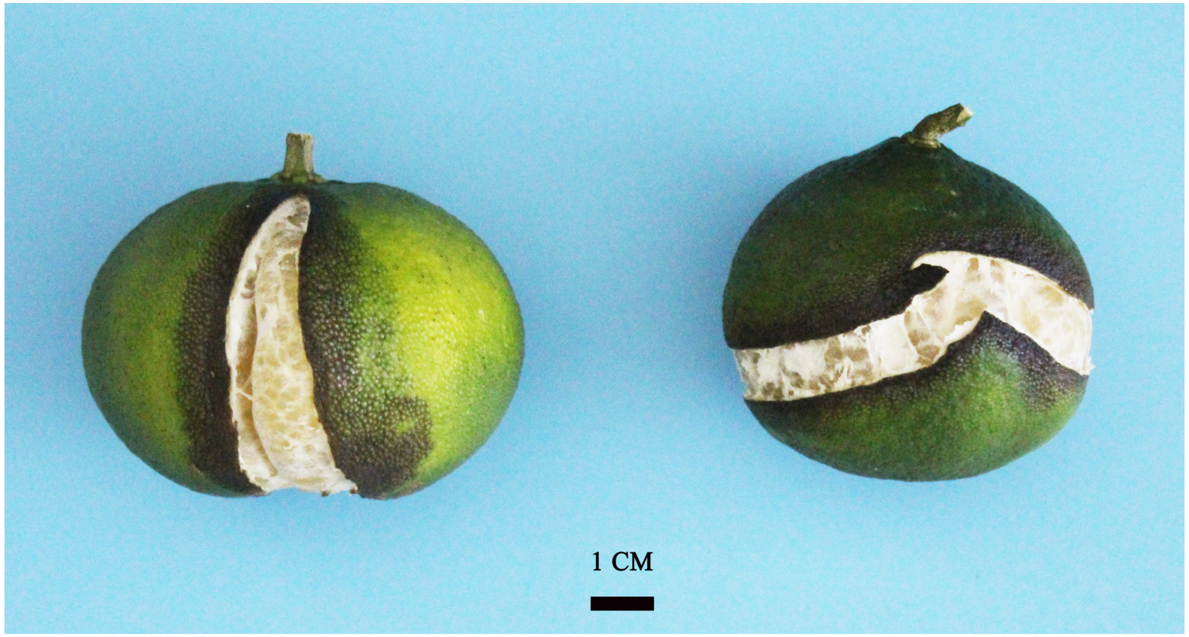

Figure S1. Two main appearance characteristics of citrus fruit split (vertical and horizontal). The samples were collected at the peak of fruit splitting during fruit development.

Tables

Table S1. The key DEMs may help to alleviate fruit splitting following treatment with potassium fertilizer.

**Metabolite**	**Description**	**Log2(FC) (Group C/A)**	***P*-value**
Amino acid[a]	gamma-Glutamylphenylalanine	2.20	0.0359
	L-Isoleucine	1.72	0.0375
	5-Hydroxytryptophol	1.58	0.0296
	Uridine diphosphate glucose	1.33	0.0337
	(±)-Tryptophan	1.35	0.0472
	Citrulline	1.27	0.0329
Hormone[a]	Cabreuvin	-30.02	0.0385
Glycoside[a]	Geranyl arabinopyranosyl-glucoside	-1.71	0.0247
	Neryl arabinofuranosyl-glucoside	-1.64	0.0019
	Agavasaponin C	34.52	0.0251
	Kaempferol 4'-glucoside 7-rhamnoside	-31.01	0.0030
	Quercetin 3-arabinoside 7-glucoside	-1.32	0.0468
Flavonoids[a]	4',5-Dihydroxy-3',5',7,8-tetramethoxyflavone	-1.24	0.0325
	7-Hydroxy-3,3',4',5,6,8-hexamethoxyflavone	-1.07	0.0085
	5,7-Dihydroxy-3',4',5'-trimethoxyflavone	-1.52	0.0152
Glycoside[b]	Arachidoside	28.81	0.0492
	Ranupenin 3-rutinoside	2.35	0.0472
	Diosmetin 7-O-beta-D-glucuronopyranoside	2.56	0.0401
	Quercetin 3-lathyroside	1.83	0.0344

Note: The letters a and b indicate that they are derived from the peel and flesh, respectively. DEMs, differentially expressed metabolites; FC, fold-change. For specific information of the fold-change in groups A and C, see Table 1.
